# Supplementary material for: Cast vote records: A database of ballots from the 2020 U.S. Election
Source: Sci Data. 2024 Nov 28;11:1304. doi: 10.1038/s41597-024-04017-1 (PMC11604945; doi:10.1038/s41597-024-04017-1)
Supplement: Supplementary file 1 — Supplementary Information [file 41597_2024_4017_MOESM1_ESM.pdf]

Supplementary Information for

"Cast vote records: A database of ballots from the 2020 U.S. Election"

This document is supplementary information for the Cast Vote Records Data Descriptor by Kuriwaki, Reece, Baltz, Conevska, Loffredo, Mutlu, Samarth, Jetter, Garai, Murray, Hirano, Lewis, Snyder, and Stewart (2024)[1].

A. Fixing Fragmented Ballots

The counties in Table S1 did not have a clear cast vote record identifier. We nevertheless paired records as one voter (one CVR ID) using a matching algorithm described in Algorithm 1. The algorithm relies on the user to supply two arguments, (1) a grouping column, `args.groupcol`, that is a condition for pages to belong to the same voter and (2) a target column, `args.targetcol`, in the CVR that can be used to match pages together. For example, if the column is the President, if two rows actually belong to the same voter, the pair must have an identical value for the `args.groupcol`, and one row will have a value for President and the other row will be blank for President.

Algorithm 1 Process Missing Values in Dataset

```
Initialize an empty set used_rows to keep track of processed rows.
Determine the total number of rows in the dataset, num_rows.
for each row current_row at index i in the dataset do
    if i is in used_rows then
        continue
    end if
    if current_row[args.targetcol] is missing a value then
        for each index j in expanding spiral around i do
            if j ≥ num_rows then
                break
            end if
            if j is not in used_rows and current_row[args.groupcol] equals data[j, args.groupcol] and data[j, args.targetcol] is not missing then
                Merge current_row with the complementary row at index j to form merged_row.
                Update data[i] with merged_row.
                Add j to used_rows.
                break
            end if
        end for
    end if
end for
```

Table S1. **Counties Where Algorithm 1 was used to re-connect pages**

County	Group Column
Alameda, California	Ballot Type
Contra Costa, California	Ballot Type
Kings, California	Ballot Type
Merced, California	Ballot Style
Riverside, California	Ballot Type
San Benito, California	Ballot Type
San Mateo, California	Ballot Type
Sonoma, California	Ballot Type
Yuba, California	Ballot Type
Denver, Colorado	Ballot Type
Eagle, Colorado	Ballot Type
Routt, Colorado	Ballot Type
Gwinnett, Georgia	Ballot Type
Baltimore, Maryland	Ballot Style
Baltimore City, Maryland	Ballot Style
Montgomery, Maryland	Ballot Style
Prince George's Maryland	Ballot Style
Butler, Ohio	Ballot Type
Champaign, Ohio	BallotStyleID
Cuyahoga, Ohio	Ballot Style
Greene, Ohio	Ballot Type
Rhode Island	Ballot Style

B. Precinct Name Standardization

Linking CVR data to other precinct-level data sources is not straightforward. The released CVR data typically contains information about the voting precinct in which each ballot was cast. However, there is no agreed-upon standard for the labeling of precincts. The precinct labels used in the CVR data often do not match those used in the official precinct-level results published by the county or state. Further, CVRs sometimes provide more detailed geographic information (commonly called "subprecinct" information) that is not included in the official precinct-level tallies.

In order to make the CVR data more useful to researchers, we manually constructed a crosswalk from the precinct labels used in the CVR data to the precinct labels in a widely-used freely-available national database of certified precinct-level 2020 General Election results published by the MIT Election Data and Science Lab (see the descriptor by Baltz *et al.*[2]). Using that crosswalk, we have appended the MEDSL precinct label to our dataset. For all linkages that we include in the data, we are completely confident that we have correctly matched precincts in the CVR and the MEDSL data. The code to construct the crosswalk can be found in our main codebase, currently at https://github.com/kuriwaki/cvr_harvard-mit_scripts/tree/main/code/01_build-returns/code.

We created this crosswalk by the following steps:

1. First, we aggregated the CVR data to the (sub)precinct level based on the precinct labels that they include. (Note, for counties such as Los Angeles, California for which the CVRs do not provide precinct identifiers, we have not attempted to place the CVRs in MEDSL precincts. Such a linkage is not in general possible, though it might be accomplished in some cases by using the set of contests included on each ballot – sometimes called the "ballot style" – to identify the precinct.) We then attempted to match our CVR-based precinct records to the MEDSL precinct data. Working county-by-county, we matched CVR precincts to MEDSL precincts based upon the reported number of votes cast for Democratic and Republican candidates for US President, US Senate, US House, State Senate, and State House. For many counties, unique exact matches for every CVR precinct could be found among the MEDSL precincts. For those counties, this set of unique exact matches was used as the crosswalk between CVR and MEDSL precincts labels.

2. For counties in which unique exact candidate vote matches could not be found for every precinct, we transformed the CVR precinct names to more closely match the format of the MEDSL precinct names. We then disambiguated cases in which a given CVR precinct's candidate vote total exactly matched more than one MEDSL precinct using the edit distance between the transformed CVR precinct name and the MEDSL precinct name by selecting the potential match having the smallest edit distance. In cases in which no exact match based on the candidate vote totals existed, we established a linkage if the transformed CVR precinct name exactly matched the MEDSL precinct name.

3. For those counties containing precincts for which neither the candidate vote totals nor the transformed precinct names could be exactly matched across the CVR and MEDSL data, potential matches were identified by the smallest sum of absolute deviations in candidate votes and confirmed by manual comparison of the CVR and the MEDSL precinct labels. Where this was not possible, no linkage was established.

4. For some counties, we determined that the CVR precinct labels included subprecinct information meaning that the CVR-based precinct-level data was at a lower level of aggregation than that reported in the MEDSL data. In those counties, we transformed to CVR precinct names to remove the subprecinct information and re-aggregated to produce CVR precinct records at the same level of aggregation as the MEDSL precincts before constructing the crosswalk using the methods described above.

MEDSL precinct labels were not applied to records for which a precinct identifier was not present in the CVR data nor to CVR precincts for which neither the names nor the vote totals established a convincing linkage between the two data sources.

In four Michigan Counties (Alcona, Clinton, Gladwin, and Missaukee) few or none of the CVR precincts could be matched to MEDSL precincts using the method described. In these four counties, the inability to link CVR precincts to MEDSL precincts appeared to be the result of irregularities in the MEDSL data. Those issues may be resolved in the future.

Fifteen other counties contained between one and five CVR precincts that could not be linked to MEDSL precincts. All of the precincts in the remaining 296 counties for which CVR precinct information was available could be linked to MEDSL precincts. In total, 28,540 CVR unique (sub)precincts were linked to 23,467 MEDSL precincts.

C. Third Party Candidates and Write-ins

Designations for third parties vary widely by state. This is compounded by the different ways counties print party on the ballot, and the types of voting machines store and record information about third party candidates and write-ins.

Howie Hawkins was the Green Party's nominee for U.S. President but the Green Party did not get ballot access in some states. Among the states that we examined, the Green Party appears to not have had ballot access in Arizona, Georgia, Pennsylvania, and Wisconsin. Some jurisdictions, e.g., in Wisconsin, do not report out Hawkins as a specific candidate in either the cast vote record or the election returns; he is lumped into simply `WRITEIN`. In Georgia, the cast vote record and the election returns report Hawkins as its own candidate, but they are still write-ins. (See https://ballotpedia.org/Georgia_official_sample_ballots,_2020 for a sample ballot, and https://bit.ly/3RYhL3A for an example of how Hawkins is reported in an election return.) In all these cases, the `party` value for Hawkins is a Write-in, not the Green Party. In contrast, New Jersey's CVRs records each and every write-in choice as a valid candidate.

It is worth noting that some of the data on `votedatabase.com` lost information about write-ins due to information loss from Excel to CSV. In some machines, notably the ES&S DS200 scanner, write-ins are scanned and can produce a cast vote record in Microsoft Excel. In these sheets, the write-in candidates are not transcribed into text but stored as an image file. However, O'Donnell instructed his collaborators to upload these data as a plain-text CSV file, which loses information in the image. In these cases, write-in votes are tracked as blank cells and excluded from our dataset. Our data, therefore, systematically misses write-in votes in many counties that use this format of cast vote record.

D. Included Counties

See `county_info.xlsx` in the Dataverse repository for the list of counties included in our data release. Figure S1 shows the geographic location of these counties.

Figure S2 shows the states in our dataset and the coverage of its population for each office. The percentage values assigned for each state indicate the total number of ballots relative to the total count of votes reported in the entire state. For example, in Texas, we have around 15 percent of the votes for the President, Congress, and state legislature. There was no election for Governor in the state.

Figure S1. **Map of Counties Included**. *(a) displays the included counties on a map of all counties in the United States. Counties shaded black are included in the data, counties shaded dark gray are part of a state that has other counties present in the data, but are not themselves present, and counties shaded light gray are neither present in the data nor is any other county from their state. (b) highlights only the states from which our data includes any counties, for easier viewing. Note that the visual area of each county is not representative of its population.*

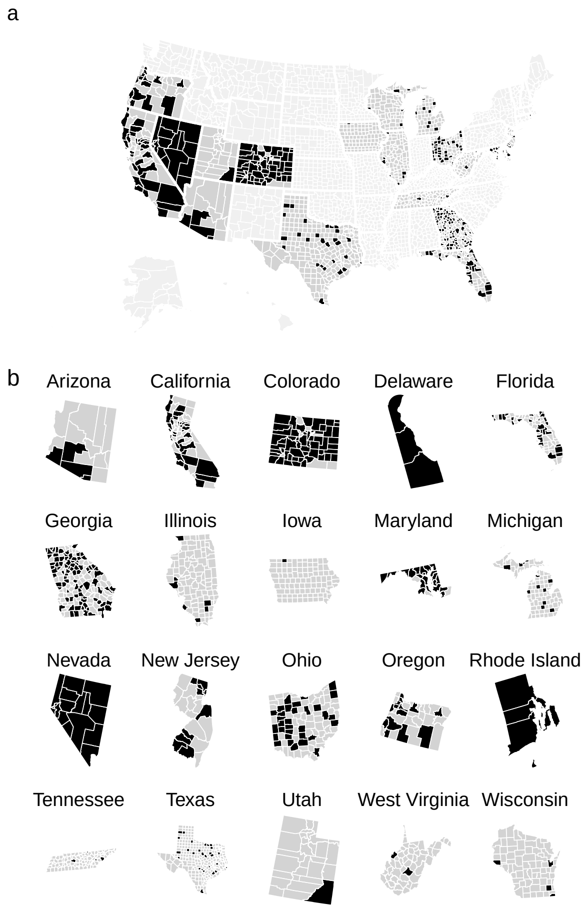

Figure S2. Coverage of CVR data. *The percentage shows the fraction of total voters contained in the CVR for that state and office. States are not shown on the map if the office was not on the ballot. States have no percentage if CVRs were not available for that state.*

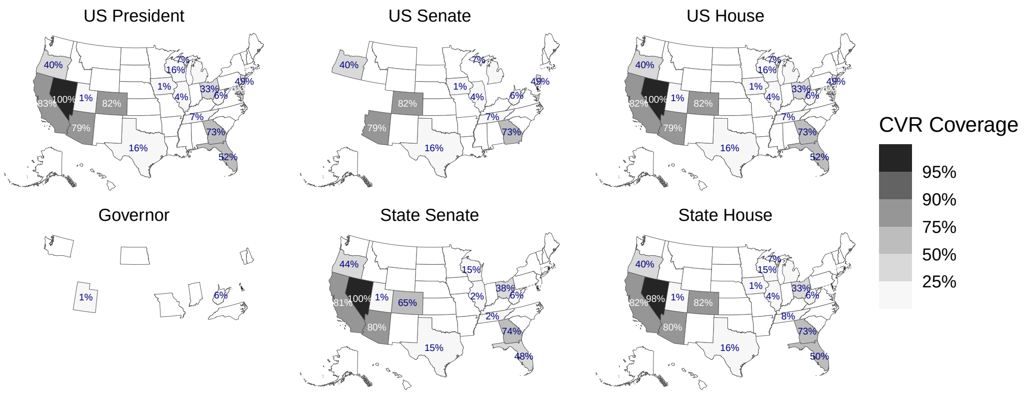

E. Extracting Summaries from the Data

In this section, we illustrate more examples of how users can extract useful summaries using our data. We open the dataset in the same manner as in the main text.

Users should use the combination of `state`, `office`, and `party` variable to identify candidates. The code below first limits to vote choices for President in Wisconsin ballots using the `filter()` command, and counts the number of records for each candidate-party collection, sorted from most frequent to least.

```
ds |>
  filter(state == "WISCONSIN", office == "US PRESIDENT") |>
  count(candidate, party, sort = TRUE) |>
  collect()
```

```
# A tibble: 8 x 3
  candidate       party      n
  <chr>           <chr>  <int>
1 DONALD J TRUMP  REP   586566
2 JOSEPH R BIDEN  DEM   442712
3 JO JORGENSEN    LBT    12544
4 WRITEIN         <NA>    2824
5 UNDERVOTE       <NA>    2674
6 BRIAN T CARROLL OTH     1748
7 DON BLANKENSHIP OTH     1448
8 OVERVOTE        <NA>     986
```

For individual voters, use the `cvr_id` variable within a state and county. This ID is a numeric variable that is defined within counties. These numbers do not in any way indicate the time in which the ballot was cast, or the personal identity of the voter. The following code extracts the vote from the voter marked with the `cvr_id` of 1.

```r
ds |>
  filter(state == "ARIZONA", county_name == "MARICOPA") |>
  filter(cvr_id == 1) |>
  select(county_name, cvr_id, office, district, candidate, party) |>
  collect()
```

```
# A tibble: 6 x 6
  county_name cvr_id office       district candidate        party
  <chr>        <int> <chr>        <chr>    <chr>            <chr>
1 MARICOPA         1 US PRESIDENT FEDERAL  JOSEPH R BIDEN   DEM
2 MARICOPA         1 US SENATE    ARIZONA  KELLY MARK       DEM
3 MARICOPA         1 US HOUSE     008      MUSCATO MICHAEL  DEM
4 MARICOPA         1 STATE SENATE 013      KERR SINE        REP
5 MARICOPA         1 STATE HOUSE  013      DUNN TIMOTHY TIM REP
6 MARICOPA         1 STATE HOUSE  013      SANDOVAL MARIANA DEM
```

This example shows that this voter split their ticket, voting for Democrats in the Presidential and Congressional race, while voting for one Republican candidate in State Senate. However, further investigation into this voter's State Senate district shows that it was uncontested. That is, with the following query,

```r
ds |>
  filter(state == "ARIZONA", office == "STATE SENATE", district == "013") |>
  count(candidate, party_detailed) |>
  collect()
```

```
# A tibble: 7 x 3
  candidate       party_detailed     n
  <chr>           <chr>          <int>
1 "KERR SINE"     REPUBLICAN     93388
2 "NOT QUALIFIED" <NA>            1852
3 "BACKUS BRENT"  <NA>             145
4 "UNDERVOTE"     <NA>           34391
5 ""              <NA>             119
6 "WRITEIN"       WRITEIN          531
7 "OVERVOTE"      <NA>              17
```

We see that none of the ballots in State Senate district 13 were for a Democrat candidate, indicating that no Democrat ran in this district.

References

1. Kuriwaki, S., Reece, M. *et al.* Cast vote records: A database of ballots from the 2020 U.S. Election, Harvard Dataverse, https://doi.org/10.7910/DVN/PQQ3KV (2024).
2. Baltz, S. *et al.* American election results at the precinct level. *Nature Scientific Data* https://doi.org/10.1038/s41597-022-01745-0 (2022).
